# The analysis of continuous data from n-of-1 trials using paired cycles: a simple tutorial

**DOI:** 10.1186/s13063-024-07964-7

**Published:** 2024-02-16

**Authors:** Stephen Senn

**Affiliations:** The University of Sheffield, Sheffield, UK

## Abstract

N-of-1 trials are defined and the popular paired cycle design is introduced, together with an explanation as to how suitable sequences may be constructed.

Various approaches to analysing such trials are explained and illustrated using a simulated data set. It is explained how choosing an appropriate analysis depends on the question one wishes to answer. It is also shown that for a given question, various equivalent approaches to analysis can be found, a fact which may be exploited to expand the possible software routines that may be used.

Sets of N-of-1 trials are analogous to sets of parallel group trials. This means that software for carrying out meta-analysis can be used to combine results from N-of-1 trials. In doing so, it is necessary to make one important change, however. Because degrees of freedom for estimating variances for individual subjects will be scarce, it is advisable to estimate local standard errors using pooled variances. How this may be done is explained and fixed and random effect approaches to combining results are illustrated.

## Introduction

This paper provides a simple tutorial on analysing continuous data from n-of-1 trials [1] using paired cycles. This design (to be described below) leads to various simple possible analyses and is an efficient way to compare two treatments on a within-patient basis where the nature of the disease and other practical considerations make this possible. The general framework that will be applied is that in which data are treated as if sampled from some hyper-population with normally distributed values. This is the usual basis for ‘parametric analysis’, which is a common device for modelling data and is known to yield (usually) similar results to an alternative framework in which the treated units are regarded as being fixed but the population is that of all possible random allocations [2, 3]. However, this correspondence works best when the sample size is large, and this is often not the case when series of n-of-1 trials are being discussed. This reservation should be noted, and in particular if the parametric analysis yields highly significant results, it may be the case that a randomisation test would be incapable of yielding similar results [4]. This is not necessarily a reason for abandoning the parametric approach. In data-poor contexts, which often apply for the study of rare diseases, accepting the necessary assumptions may be the lesser of two evils. Nevertheless, the limitation should be born in mind.

The objectives of the tutorial are to provide simple justifications and instructions for various possible analyses of such trials and also explain for which purposes they are suited. Use of algebra is kept to a minimum, and graphical and tabular representation of data and analyses are stressed.

For readers who require more technical detail, a general model for data from N-of-trials is presented and discussed in an appendix. It is explained how the way in which the overall treatment effect is regarded, either as a mean effect for the subjects studied or as a mean effect of the hypothetical population of subjects of whom they might be considered to be a random sample, will affect the way that analysis proceeds.

### The design

A common design for n-of-1 trials comparing two treatments is to organise allocation in such a way that within any given pair of periods each treatment is used once [5–7]. Such pairs of periods have been referred to as *cycles* [8]. A possible scheme for a design in three cycles is given in Table 1. Patients would then be allocated at random to one of the eight possible sequences.

**Table 1:**
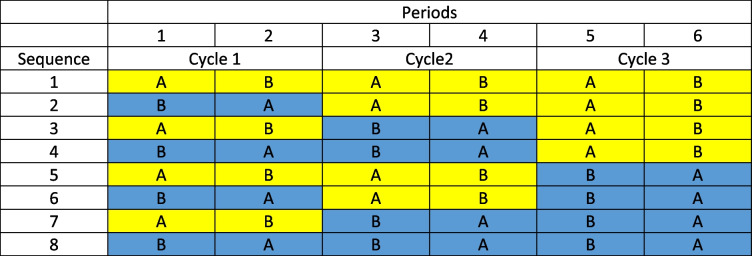
Set of sequences for a design using six periods arranged in three cycles. Pairs with A followed by B are shaded yellow. Pairs with B followed by A are shaded blue

In general, if there are *k* possible cycles in which patients can be treated, there will be 2^k^ possible sequences. A *canonical set* of possible sequences can be constructed as follows using the basic pair AB and BA. When moving between successive sequences in a list of sequences, for cycle 1, switch AB and BA after every sequence. For cycle 2, double the number of sequences before switching. For each successive cycle, double the sequences before switching.

This design is relatively simple to organise and efficient and lends itself to various simple analyses. As regards organisation, a simple way to implement randomisation to sequences is just to randomise patients independently for each cycle. As regards the second, the close temporal control that is offered by randomising in pairs makes it efficient. It could be argued that if carry-over is likely and one wishes to guard against it, various other designs might be preferable, but the solutions these offer depend on implausible modelling assumptions, and the best advice as regards carry-over is to ensure adequate washout between treatments, if necessary limiting measurement of the effect of each treatment towards the end of periods in which they are given [9]. As regards analysis, it is the purpose of this note to explain how this may be achieved. For advice on reporting n-of-1 trials, see the CENT statement [10].

### Illustrative data for analysis

N-of-1 trials lend themselves to addressing a number of different questions that might arise naturally in connection with studying the effects of treatments [11]. The questions are as follows:

Q1. Was there an effect of treatment in the trials?

Q2. What was the average effect of treatment in the trials that were run?

Q3. Was the treatment effect identical for all patients in the trials?

Q4. What was the effect for individual patients in the trials?

Q5. What will be the effect of treatment when used more generally (in future)?

The suggested analyses will be organised in terms of these questions. We shall use the simple simulated data that were presented in Araujo et al. [8] to illustrate these analyses.

It is supposed that a trial in asthma has been carried out comparing two treatments, A and B, each given as a single dose. Twelve patients have been randomised in pairs of cycles as described above. The first ten have completed all three planned cycles of treatment. However, patient 11 has only completed two cycles of treatment and patient 12 has only completed 1. This has been done to illustrate a complication in analysis that may arise in practice. We thus have data from (10 × 3) + 2 + 1 = 33 cycles and therefore from 2 × 33 = 66 episodes. In all the analyses that follow, we shall assume that the fact that some values are missing is uninformative and that reasonable inferences may be based on the values that remain.

The results are measurements of forced expiratory volume in one second, FEV_1_, in mL taken 12 h after treatment. The data are presented in Table 2 sorted by treatment within cycle (that is to say A then B). The period in which A or B was administered is given within Table 2, and this reflects the randomisation used. The data are also available to download from https://journals.plos.org/plosone/article?id=10.1371/journal.pone.0167167#sec009. Note, however, that those data include values for cycles 3 from patients 11 and 12 and cycle 2 from patient 12, which are assumed missing here.

**Table 2:** A simulated trial in asthma. Twelve patients have been randomised in three cycles to treatment A followed by B or B followed by A. The table gives the periods in which the patients received A or B and the FEV_1_ in mL below. For example, patient 1 received treatment A in periods 1, 3, and 6 and treatment B in periods 2, 4, and 5

			Treatment			
Patient	A	B	A	B	A	B
1	1	2	3	4	6	5
	2394	2686	2515	2675	2583	2802
2	2	1	3	4	6	5
	2746	2726	2592	2867	2743	2742
3	1	2	3	4	6	5
	2668	2560	2542	2584	2491	2737
4	1	2	3	4	6	5
	2397	2696	2411	2895	2499	2760
5	2	1	3	4	5	6
	3179	3221	2952	3096	2600	3192
6	1	2	4	3	5	6
	2643	2496	2759	2847	2651	2860
7	1	2	3	4	5	6
	2678	2843	2492	2763	2801	2890
8	2	1	3	4	5	6
	2887	2862	2875	3083	2689	2967
9	2	1	3	4	6	5
	2490	2841	2648	3044	2688	2914
10	2	1	3	4	6	5
	2268	2576	2413	2493	2344	2699
11	2	1	4	3	6	5
	2617	2923	2629	2832		
12	1	2	4	3	5	6
	2627	2759				

**Fig. 1:**
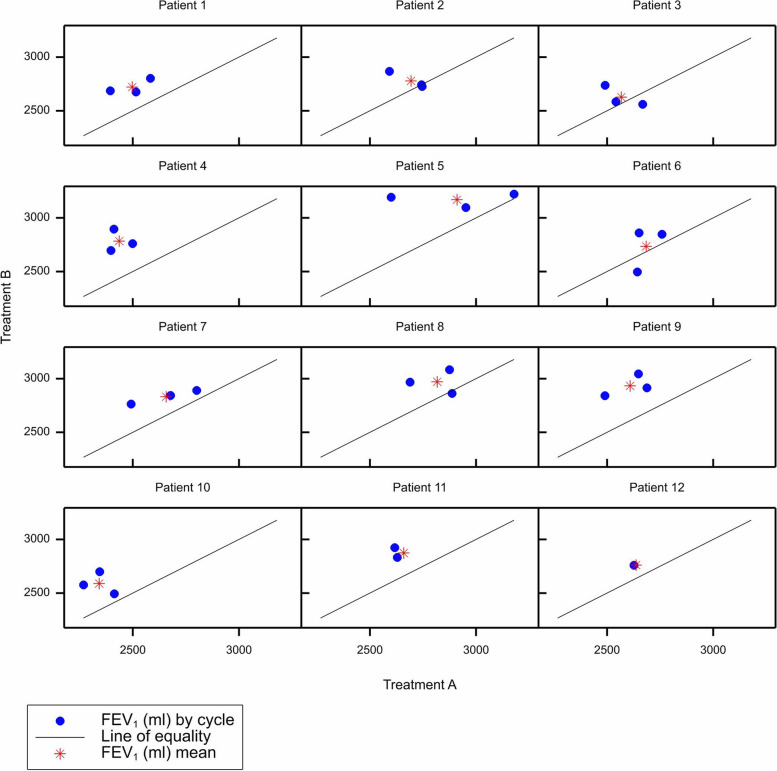
Trellis plot of the results from the simulated example

A useful plot of the data is given in Fig. 1, which is a *trellis plot*. Each window represents the results for a given patient. The result for each cycle is represented by a blue circle plotting the value under B (*Y* axis) against that under A (*X* axis). The diagonal line represents equality between the two treatments. The average values over all cycles are represented by red asterisks. It is noticeable that the blue circles are generally above and to the left of the line of equality suggesting that B has a bigger effect than A.

### Demonstrating that there can be a difference between treatments

Q1, ‘was there an effect of treatment in the trials?’, leads to a very simple analysis. The relevant null hypothesis is that there is no difference between treatments *for any of the patients*. If that is the case, under the null hypothesis, it does not matter which patient is studied; the result may be expected to be the same. This renders the *differences* between A and B as being independent over patients *by hypothesis*. That being so, we can carry out a matched pair analysis on the 33 cycles.

The data have been reduced to differences by cycle and patient and are presented in Table 3. These differences can be analysed by a one-sample *t*-test for which the statistics in Table 4 are produced.

**Table 3:** Differences (treatment B − treatment A) per cycle arranged by patient

Cycle	1	2	3
Patient			
1	292.0	160.0	219.0
2	− 20.0	275.0	− 1.0
3	− 108.0	42.0	246.0
4	299.0	484.0	261.0
5	42.0	144.0	592.0
6	− 147.0	88.0	209.0
7	165.0	271.0	89.0
8	− 25.0	208.0	278.0
9	351.0	396.0	226.0
10	308.0	80.0	355.0
11	306.0	203.0	*
12	132.0	*	*

**Table 4:** Summary statistics to perform a one-sample *t*-test based on differences per cycle

Statistic	Value	Explanation
*n*	33	Number of cycles
Mean	194.55 mL	Mean of the 33 cycle differences
Variance	26188 mL^2^	Sample variance of the 33 cycle differences
SD	161.8 mL	Standard deviation = √variance
SE	28.17 mL	Standard error = SD/√*n*
DF	32	Degrees of freedom = *n*-1
*t*	6.91	*t*-statistic =194.55 mL/28.17 mL
*P*-value	< 0.001	Probability under H_0_ a *t*-statistic with 32 DF will be ≥ 6.91 or ≤ − 6.91

Note that although independence is guaranteed under H_0_ by hypothesis, the same is not true under many alternative hypotheses. For example, it might be the case that some subjects would show a large treatment effect but some would show no effect at all. It might be interesting to develop a test that was powerful for this sort of alternative hypothesis (for a suggestion for parallel group trials, see Conover and Salsburg [12]). However, a simple analysis that does remove the treatment-by-patient interaction can be constructed by estimating the variance patient by patient comparing the cycle differences to the mean for that patient. This is discussed in the next section.

### Putting bounds on the mean effect for the patients studied

We now consider how we may answer Q2 ‘what was the average effect of treatment in the trials that were run?’. Note that if we decide that this effect is not zero, we have also answered Q1. This issue will be discussed subsequently. For the moment, we address an analysis to answer Q2.

The critical value at the 5% level two-sided for a *t*-statistic with 32 degrees of freedom is 2.037. If this is multiplied by the standard error, the product is 2.037 × 28.17 mL = 57.38 mL. If this is subtracted and added to the mean of 194.5 mL, then we obtain a 95% confidence interval for the mean effect (to one decimal place) of (137.2 mL, 251.9 mL).

This particular calculation can be criticised. Whereas it is reasonable to assume by hypothesis that the treatment effect is constant for all patients, when we are testing that this effect is zero for them all, as soon as we allow that the effect is *not* zero, it becomes plausible that it might vary from patient to patient, as discussed above. If we regard the patients as being fixed, that is to say that we are only making a statement about these patients, then we could claim that this source of variation would not contribute to the treatment estimate changing were we to repeat the experiment. However, it will contribute to the overall estimate of variation that we have used.

This source of variation can be eliminated by constructing variance estimates patient by patient. The calculations are given in Table 5.

**Table 5:** Intermediate calculation to estimate the common within-patient variance. Note that the units of variances and sums of squares are mL^2^ of FEV_1_

Patient	DF	Variance	Sum of squares
1	2	4372.3	8744.7
2	2	27260.3	54520.7
3	2	31572.0	63144.0
4	2	14233.0	28466.0
5	2	85601.3	171202.7
6	2	32767.0	65534.0
7	2	8356.0	16712.0
8	2	25166.3	50332.7
9	2	7758.3	15516.7
10	2	21636,3	43272.7
11	1	5304.5	5304.5
12	0	0.0	0.0
Total	21		522750.5

Here, the column labelled *DF* gives the degrees of freedom patient by patient and is equal to the number of cycles minus 1. The column labelled *Variance* gives the *local* estimate of the variance of the differences (B-A) patient by patient. For patient 12, the value is zero since the patient was only studied in one cycle and hence there is only one difference. The column headed *Sum of Squares* is obtained by multiplying the variance by the degrees of freedom. The overall sum of squares is 522,750.5 mL^2^, and if this is divided by the total DF, 21, we obtain 24,893 mL^2^, which is thus our estimate of the variance on the assumption that variability does not vary from patient to patient.

The consequent calculations are summarised in Table 6.

**Table 6:** Summary statistics to perform a one-sample *t*-test based on differences per cycle with the patient by treatment interaction removed from the variance estimate

Statistic	Value	Explanation
*n*	33	Number of cycles
Mean	194.55 mL	Mean of the 33 cycles
Variance	24893 mL^2^	Sample variance of the 33 cycles
SD	157.8 mL	Standard deviation = √variance
SE	27.47 mL	Standard error = SD/√*n*
DF	21	Degrees of freedom = *n*-1-11
t	7.08	*t*-statistic =194.55 mL/27.47 mL
*P*-value	< 0.001	Probability under H_0_ a *t*-statistic with 21 DF will be ≥ 7.08 or ≤ − 7.08

Note that compared to the naïve test of the ‘Demonstrating that there can be a difference between treatments’ section, we have gained some reduction in variance at the expense of losing some degrees of freedom. The former will increase the power of the test, but the latter will reduce it. Thus, even if our objective is answering Q1, we might do better using the test for which the treatment-by-patient interaction has been removed from the error term. In general, it is not possible to say in advance of examining data which will be more powerful. If we can believe in the complete homogeneity of the treatment effect, the test of the ‘Demonstrating that there can be a difference between treatments’ section will prove so. In the presence of considerable heterogeneity, the test of this section may do so.

However, there is another issue that arises. If we do not regard the patients as fixed, then we have not reflected the variation from patient to patient enough, since our estimate is based on using cycles as the unit of inference rather than patients. Furthermore, the mean over all cycles will not weigh the patient means equally because not all patients have been observed as often: the mean of 33 cycle differences will not be the same as the mean of 12-patient differences. Note that considerations of this sort raise difficult issues. If we accept that the effect of treatment varies for different patients, then it would seem logical that this component of variation (the variation of the true effect from patient to patient) should contribute to our uncertainty about the true average effect more widely, since we accept that different patients could have given us a different answer. The problem is, however, not only that we cannot regard patients recruited in a clinical trial as being a random sample of some target population we might have in mind but also that it is difficult to establish of what population they *could* be regarded as being a random sample. A possible strategy is to perform the analysis as if the patients were such a sample of such a population but to recognise that the attendant uncertainty will be underestimated by such an analysis.

We now put these concerns aside for the moment and consider an analysis that uses patients as the unit of inference.

### Putting more general bounds on the treatment effect

One way of proceeding is to reduce the differences to a mean per patient and then perform an analysis using these 12-mean differences as our raw input. The data are presented in Table 7. We shall ignore the column labelled ‘Standard error’ for the moment (we shall use this later). Instead, we just base our analysis on the 12 *per patient estimates*.

**Table 7:** Summary statistics per patient that may be used for various analyses

Per patient estimate	Standard error
223.7	91.09
84.7	91.09
60.0	91.09
348.0	91.09
259.3	91.09
50.0	91.09
175.0	91.09
153.7	91.09
324.3	91.09
247.7	91.09
254.5	111.56
132.0	157.77

If we carry out a one-sample *t* analysis on these values, we can summarise the results as in Table 8.

**Table 8:** Summary statistics to perform a one-sample *t*-test based on differences per patient

Statistic	Value	Explanation
*n*	12	Number of patients
Mean	192.74 mL	Mean of the 12 patient means
Variance	9895 mL^2^	Sample variance of the 12 patient means
SD	99.48 mL	Standard deviation = √variance
SE	28.72 mL	Standard error = SD/√*n*
DF	11	Degrees of freedom = *n*-1
t	6.71	*t*-statistic =192.7 mL/28.72 mL
*P*-value	< 0.001	Probability under H_0_ a *t*-statistic with 32 DF will be ≥ 6.71 or ≤ − 6.71

The end result is very similar to that reached before. It is not surprising that the mean is scarcely different. The fact that the standard error is similar, however, reflects the fact that *for this particular example* the variation in effect from patient to patient over and above that to be expected by the random variation from cycle to cycle is small. Nevertheless, the analysis is conceptually different to that previously provided as it has greater relevance to a different question: *what can one say about the mean effect in general, not just for patients studied*. This is a form of Q5 considered above, but note that the previous discussion in the ‘Putting bounds on the mean effect for the patients studied’ section highlighted some inferential problems and that the tentative nature of such answers should not be forgotten. We shall revisit this question in the ‘Meta-analytic approaches’ section.

There are now 11 degrees of freedom, and the critical value for the *t*-statistic is now slightly larger at 2.201. We thus have 2.201 × 28.72 mL = 63.21 mL as the value that has to be subtracted from and added to the mean to get lower and upper 95% confidence limits. The resulting 95% confidence interval is (129.5 mL, 255.9 mL).

## Meta-analytic approaches

A set of n-of-1 trials which we have been considering is analogous to a collection of results from independent clinical trials, which might be summarised in a meta-analysis. There is an extensive theory of how such results should be analysed [13–15], and software routines exist within many major statistical packages that may be used to perform a meta-analysis. This means that tools are available that may be simply adapted to perform the analysis of a set of n-of-I trials.

There is one important change in data-preparation that is, however, necessary. Standard meta-analytic approaches assume that the standard errors used to calculate the weights are themselves calculated without error. This is, of course, not true. Estimated standard errors are random variables, not known parameters. However, if the associated degrees of freedom are reasonably large, this assumption does not matter. For n-of-1 trials, however, there are typically few degrees of freedom per patient. In our example, there are no more than two per patient. Naively estimating the variances independently is unwise [16, 17]. It is better to use a pooled variance to do so.

Thus, we impose an assumption that the within-patient *variation* between estimates per cycle is constant across patients. We then proceed to estimate the variance.

For this purpose, we can use the approach illustrated in Table 5 and Table 6. For each patient, the degrees of freedom are calculated as the number of cycles in which they were treated minus one. The values are shown in column two of Table 5. The sample variance of the estimated treatment effect for each patient is calculated and given in column three. The product of the values in columns two and three gives the sums of squares (corrected by the mean), which is shown in column four (if the available statistical software package has a standard function available for the *corrected sum of squares*, it may be easier simply to calculate column four directly). The sum of the values in column four is 522,750.5 mL^2^. Dividing the total sum of squares by the total degrees of freedom, 21, yields an estimated variance of 24,892.9 mL^2^, and the square root of this is 157.77 mL.

Note that since patient 12 was only treated in one cycle, it is impossible anyway to estimate a variance for them. However, using the data from other patients, we assume that the estimated standard deviation for them is the same as for all patients and is thus 157. 77 mL. Since the estimate for this patient is only based on one cycle, the standard error for them is the same as the standard deviation, since, trivially, $\frac{157.77\ mL}{\sqrt{1}}=157.77\ \rm{mL}$. In general, if a patient was treated in k cycles, we have $SE=\frac{s}{\sqrt{k}}$, where *s* is the estimated pooled standard deviation (157.77 mL for this example). For patient 11, we have *k = 2*, and for patients 1 to 10, *k = 3*. Substituting these values of *k* yields the standard errors given in Table 7.

**Fig. 2:**
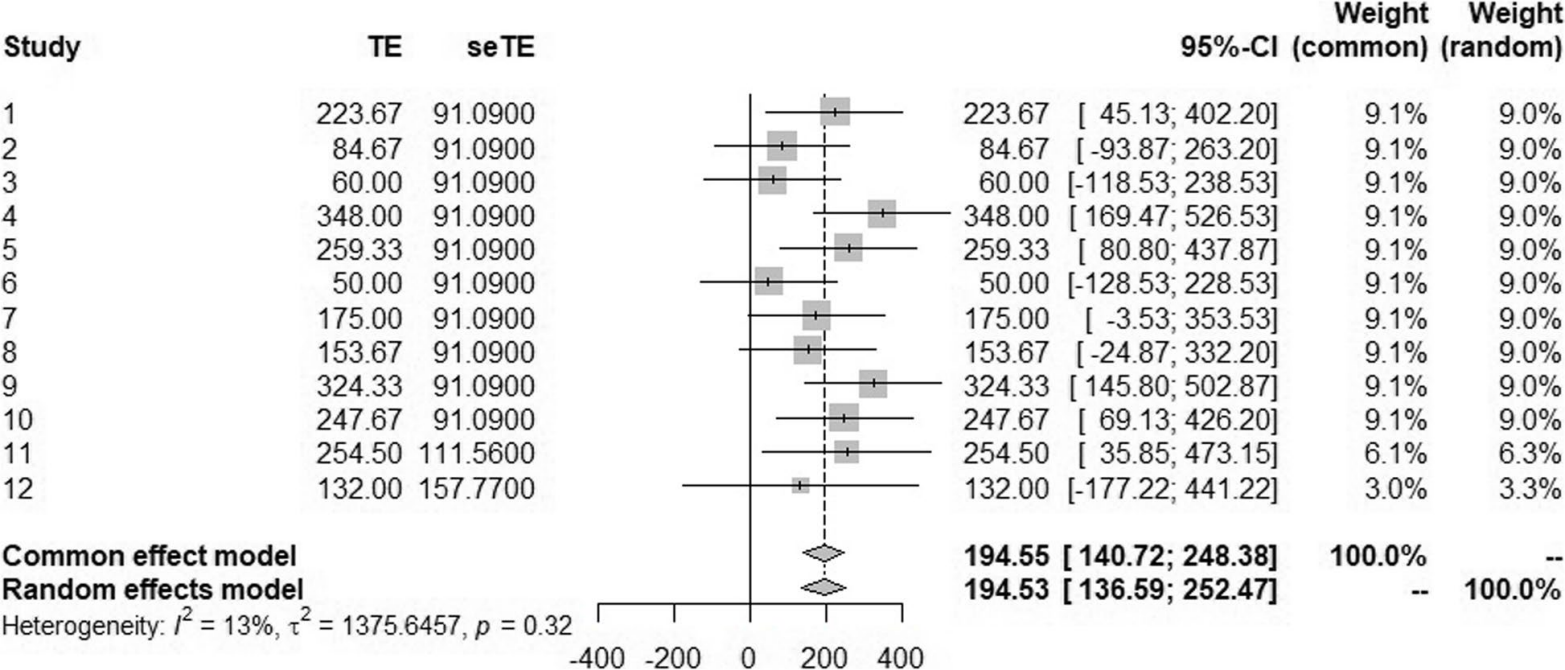
Results of analysis using the *meta* package

We can now apply standard meta-analytic approaches to the data in Table 7. There is a wide choice of packages to do this. Here, we illustrate the analysis using the *meta* package of Guido Schwarzer’s [18]. The results of using the *metagen( )* and *forest( )* functions are displayed in Fig. 2.

This provides both a fixed and a random effects analysis. For the latter, there are a number of possible methods, and the DerSimonian and Laird [19] approach has been chosen. For this example, the results of fixed and random effects analyses are very similar. Furthermore, the point estimate of 194.55 mL is identical to that reached for the matched pairs analysis of the 33 cycles. This is no coincidence. Since the standard errors patient by patient have been calculated using the same variance, the difference between them merely reflects the numbers of cycles for which information was obtained. The *metagen( )* function is a *generic inverse variance meta-analysis* function. It weighs results proportionately to the inverse of the square of the standard error, that is to say proportionately to the number of cycles.

The standard error is different however. This is based on 21 degrees of freedom rather than 32. The extent to which results vary from patient to patient has been removed from the estimate of the variance. The difference is 11 degrees of freedom, and these are the degrees of freedom that correspond to the treatment-by-patient interaction. As has been pointed out elsewhere, this point is frequently misunderstood [20]. More generally, both a fixed and random effect interaction fit a treatment-by-trial interaction (in this case, the analogy of *trial* is *patient*). It is what they *do* with it that makes the difference.

The estimate of the treatment-by-trial interaction may be used to answer Q3: ‘Was the treatment effect identical for all patients in the trials?’ The relevant variance is given as *τ*^2^ = 1376 (to the nearest square mL) and the associated *P*-value as *p* = 0.32. Thus, using the conventional threshold of 5% for statistical significance, the result is not ‘significant’. However, this non-significance does not prove that there is no heterogeneity and, furthermore, whether or not there is heterogeneity is not the issue in choosing between fixed and random effects analyses. It is the purpose which guides the choice [20].

The random effects meta-analysis estimate has a slightly wider confidence interval. This is because it provides an estimate of the treatment effect that would apply were it the case that the patients that have been studied were no longer fixed but could be regarded as a random sample from a wider but ‘similar’ population. Thus, the differences in effect that the interaction measures are no longer regarded as being fixed but as having values that might vary from one occasion to another. Thus, this uncertainty is incorporated in the confidence intervals. In favour of the random effects analysis is the fact that it addresses a more important question. Against it is the fact that, to answer this question, strong assumptions (the similarity of patients studied with those in the target population) have to be made.

### Estimates of effects for individual patients

Q4 in our list of five questions was ‘What was the effect for individual patients in the trials?’ It may surprise some that superior estimates of the effects from individual patients can be obtained by also using the results from others. However, a little reflection shows that using results from others is exactly what happens when data from parallel group trials provide predictions of the effect of treatments. Thus, we use often estimates of average effects to predict effects for individual patients. Therefore, a series of n-of-1 trials will provide two sorts of information for a given individual, namely *personal* and *global*, the former only using a given patient’s data and the latter all the data. Each of these is an unbiased estimate of the effect for a patient, and they may be combined to produce a so-called *shrunk* estimate as follows

$$shrunk=w\times personal+\left(1-w\right) global,$$

where *w* is a weight between 0 and 1. The greater the value of *w*, the more attention we pay to the information from the given patient. The estimate is referred to as *shrunk* because the result will lie between personal and global and so may be regarded as having shrunk towards the latter compared to the former. An alternative term is *best linear unbiased predictor* (BLUP) [21].

Just as we combine information from a meta-analysis by weighing the trial proportionately to the inverse of the variances of their estimates, we weigh these two sorts of information inversely according to their variances.

**Fig. 3:**
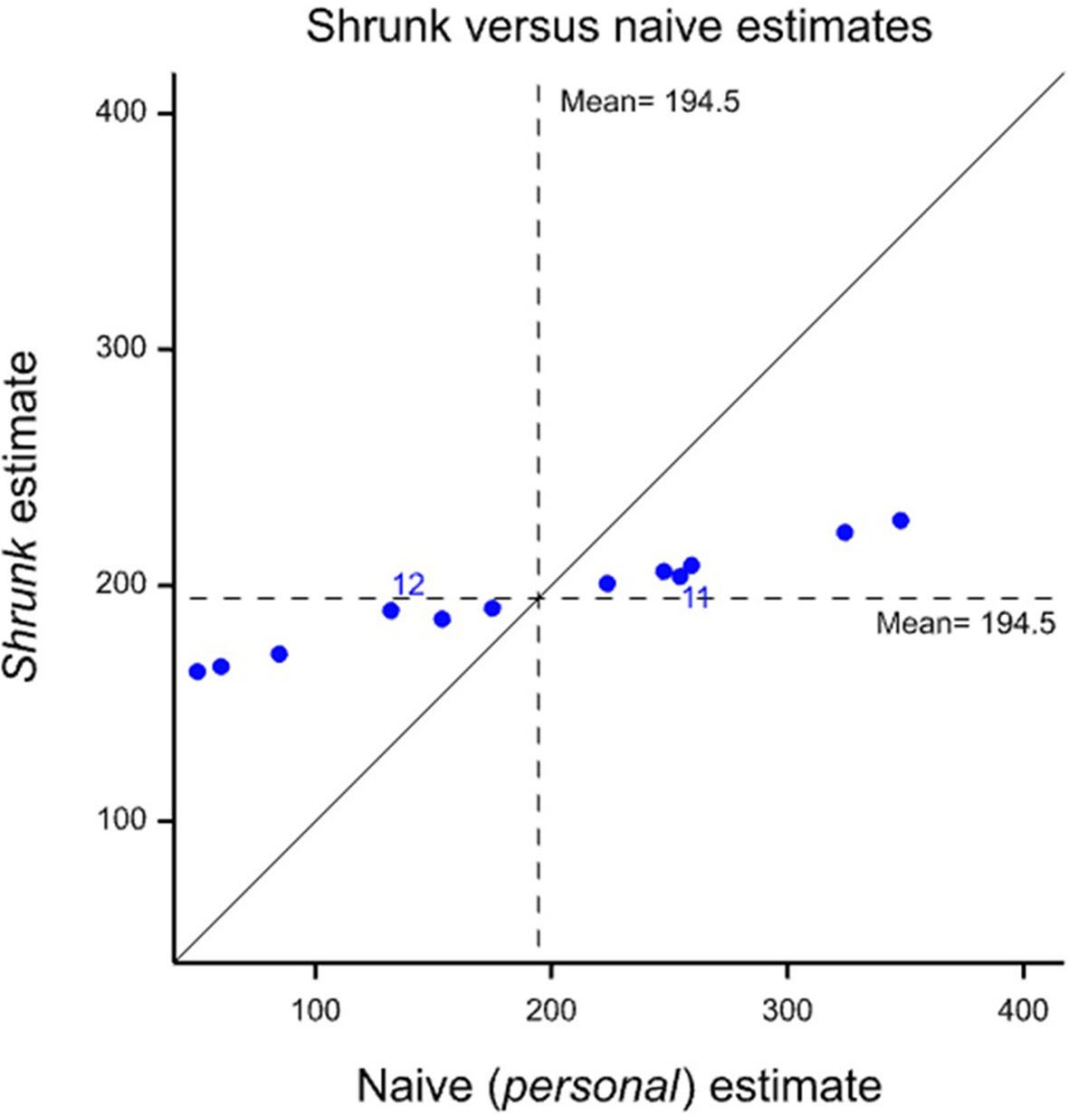
Shrunk estimates for FEV_1_ in mL based on a weighted combination of global and personal estimates versus the naïve estimate based on personal information only. The diagonal line is the line of equality

A plot of the shrunk estimates is provided in Fig. 3, which exhibits strong shrinkage. The reason that this is so is because there is little evidence of differences in the effect of treatment from one patient to another, what observed differences there are being largely due to within-patient variation, that is to say random variation of observed effects from cycle to cycle. For patients 1 to 10, the degree of shrinkage is the same, so that their points line on a straight line. Patients 11 and 12 are labelled because they have different (stronger) shrinkage, since their results are based on two cycles and one cycle respectively rather than on three.

We do not need to go into the theory of this more deeply; it is covered, for example, in the paper by Araujo et al. [8], already cited, and also in Senn (2019) [22]. Fortunately, this sort of question is addressed in various meta-analytic packages. For example, the *metafor* package [23] within R has a *blup( )* function that will do this. It is, of course, necessary to have prepared the data in the way described at the beginning of this section.

### Linear mixed effect and non-linear mixed effect models

Most of the analyses shown so far can be regarded as special cases of so called *linear mixed effects models* [21, 24, 25]. For example, an alternative if we have a model with treatment, patient, and cycle within patient as fixed effects, will produce an analysis equivalent to the matched pairs approach using the 33 cycles. If, in addition, we declare the treatment by patient interaction as random, an analysis that is very similar to the random effects meta-analysis will be produced. Such models provide a powerful, flexible framework for analysis but do require greater statistical skill in their handling and are not covered in this simple tutorial. For more information about their application to n-of-1 trials, see the papers by Araujo et al. [8], Zucker et al. (2010) [7], and Van Den Noortgate and Onghena [26].

For other outcomes, for example binary outcomes, non-linear mixed effect models may be used. Their application to crossover trials is covered in Senn (2002) [9], Senn (2021) [27], and Jones and Kenward (2015) [28].

### Analysis when there is only one patient

The techniques discussed so far are applicable when results can be obtained from a number of patients, which means that these results can be combined not only for the purpose of examining average effects of treatment but also for the purpose of producing superior shrunk estimates for individual patients. It is sometimes the case, however, that the rarity of the disease or other practical difficulties mean that very few patients, and in the limit only one, can be recruited.

Given the possibility of treating the patient for many cycles, a reasonable analysis could still be carried out, although if degrees of freedom are few, there might be an advantage in abandoning the idea of pairing in cycles and using a completely randomised design. Such a design was famously considered by RA Fisher [29] in testing Muriel Bristol’s ability to taste whether the tea she was given has milk in first or tea in first. Eight cups were used, and this gives $\frac{8!}{\left(4!4!\right)}=70$ possible sequences. Using four pairs of cups would only yield 2^4^ = 16 possible allocations and make guessing all cups less impressive. However, this approach would lead us beyond the theme of this paper and will not be considered here. For possible approaches to this sort of trial, see, for example, the book by Dugard et al. [4].

However, if a design in paired periods is used and if only a few cycles are available, a severe difficulty presents itself. Suppose that, as was the case in our simulated example, only three cycles can be used. In that case, not only will the mean effect be estimated poorly, the variance of the effect will be estimated extremely poorly, since only two degrees of freedom will be available. This is what might be called a matter of *second-order efficiency* [30]: the effect is on the estimate of variability not on the variability of the estimate. This has a catastrophic effect on calculating confidence intervals or significance. For the simulated example, by estimating the variance from all the patients, we had a variance estimate with 21 degrees of freedom. The 97.5% quantile on the *t*-distribution with 21 degrees of freedom is 2.080. On the other hand, with only two degrees of freedom, it is 4.303, more than twice as large. Hence, other things being equal, confidence intervals for treatment effects would be more than doubled were we to use the local (to each patient) values for estimating the variance.

One possibility is to try and use an external estimate for the variance of the effect, even if it is accepted that the estimate of the effect itself must be limited to the patient. This is very much in the spirit of post-hoc ANOVA tests, where variances are often pooled across treatments even if only two of them are being compared. This habit originated in agriculture where degrees of freedom are scarce and, not always logically, is often used in multi-armed parallel group trials, pooling the variance from all treatments, even when only two are being compared, despite the fact that degrees of freedom are abundant [11, 31].

Even if a treatment is being trialled for the first time, it may be the case that the disease has been studied previously. One solution would be to use a suitable variance estimate from such studies to calculate the standard error for the n-of-1 trial. Care needs to be taken to match like with like. It has to be a within-patient variance, and a trap must be avoided. The variance of the difference between two observations on a given subject is *twice* the within-subject variances as usually defined by statisticians. It might be appropriate to cap the number of degrees of freedom for such a historical variance at some relatively low number, say 10, even where many subjects have been studied and pool accordingly with the data from the n-of-1 trial.

**Fig. 4:**
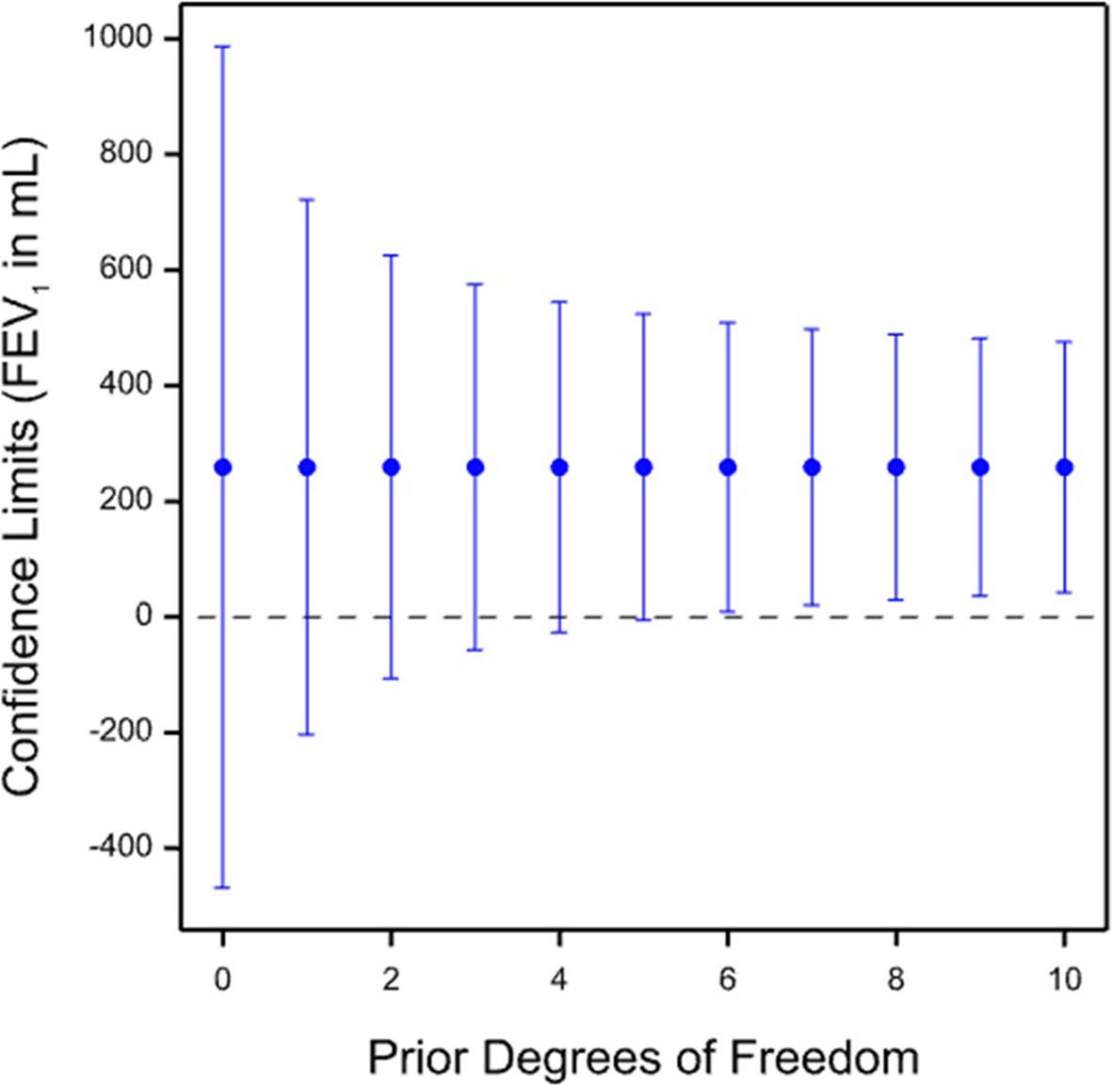
Illustration of technique of pooling a prior variance with the variance from a given patient (in this case patient number 5). The 95% confidence limits are shown. Information from the other patients is assumed to be available, and various possible weights in terms of ‘prior degrees of freedom’ are considered. The point estimate is unaffected, but depending on prior degrees of freedom assumed, the estimated variance and the critical value of the *t*-distribution will change

Such an approach is illustrated in Fig. 4. It is assumed that only patient number 5 of those previously considered is being measured. However, information on variability of results is available from other historical patients (here, the data from the remaining 11 patients has been used). These data are combined with those from patient 5 to form a weighted variance, where the weights are the two degrees of freedom available for patient 5 and the assumed ‘prior degrees of freedom’ varying from 0 to 10 for the remaining patients (note that this is a deliberate choice and is not the same as the actual degrees of freedom used in estimating this prior variance). The resulting ‘posterior degrees of freedom’ will be the sum of the two and thus vary from 2 to 12. The critical value of the *t*-distribution is calculated accordingly, as is the standard error and hence the confidence limits are obtained.

If the prior degrees of freedom are 0, then the result is equivalent to just using the data from patient 5. Prior information is not used to calculate the point estimate, which thus remains unchanged. The variance will change, and this might increase or decrease depending on whether the variance for the patient under consideration is smaller or larger than that from the historic data. Here, patient 5 had a larger than average value. Whether the variance and hence the standard error increases or reduces, the critical value of the *t*-distribution for calculating the 95% limits will shrink towards the asymptotic value of 1.96 that applies to the normal distribution. For two (posterior) degrees of freedom, the value is 4.30, and for 12, it is 2.18.

Of course, this is all very speculative, but desperate remedies may be needed when data are scarce.

## Conclusions

N-of-1 trials encourage us to look at treatment effects at the lowest level, that of patients themselves. Of course, this is the level at which decisions are made, and so, ideally, it is the level at which we should like to estimate effects of treatment. Nevertheless, random variability will still affect our estimates, and combining local and global information will often lead to worthwhile improvements in precision. The scarcity of data may make some compromise as regards standards inevitable, but what should not be compromised are the standards employed in explaining what has been done. Assumptions should be stated, and the aim should be to make it as clear as possible what choices have been made and how they have been implemented.

It is hoped that this tutorial has succeeded in explaining how this may be done.

### Software

Example programs in SAS®, R®, and Genstat® can be found on the DIAMOND website [32]. The 24th edition of Genstat® has a number of procedures for analysing n-of-1 trials [33]. See also Artur Araujo’s report [34] on analysing n-of-1 trials for useful code in R.

## Data Availability

Code in Genstat®, SAS®, and R® is available on request from me.

## Appendix

A general model for outcomes from n-of-1 trials arranged in cycles can be expressed as follows.

1 $${Y}_{ir s}={\lambda}_i+{\beta}_{ir}+{\varepsilon}_{ir s}+{Z}_{ir s}{\tau}_i,$$

where *Y*_*irs*_ is the measured outcome for occasion *s*, *s* = 1, 2 of cycle *r*, *r* = 1, 2…*k*_*i*_ for patient *i*, *i* = 1, 2⋯*n*. Here, *λ*_*i*_ ∼ *N*(*Λ*, *ϕ*^2^)is a random effect for patient *i*, *β*_*ir*_ ∼ *N*(0, *γ*^2^) is a random effect for cycle *r*within patient *i*, *ε*_*irs*_ ∼ *N*(0, *σ*^2^) is a random error term for occasion *s*of cycle *r* for patient *i*, and *τ*_*i*_ ∼ *N*(*Τ*, *ψ*^2^) is a random treatment effect for patient *i*, with ${Z}_{irs}=-\frac{1}{2},\frac{1}{2}$, depending on whether the patient was assigned A or B on that occasion in that cycle. All stochastic terms are assumed independent of each other.

It is worth drawing attention here to a potential point of confusion. If we study the variation of the difference between treatments A and B for a given patient, the variance of these differences will be expected to be 2*σ*^2^ because each within cycle difference has a contribution from two errors, *ε*_*ir*1_, *ε*_*ir*2_. Because in a matched pairs analysis, 2*σ*^2^is estimated directly, but in a linear model, one would estimate the variance of the *ε*_*irs*_terms, which is *σ*^2^, there is a danger that readers may misunderstand what an author means by referring to a within-cycle variance. If variance terms are picked up from a paper for planning purposes, there is a danger of miscalculation of the necessary sample size by either a factor of two or of one half. The moral is it is best to be explicit, and indeed, in an earlier version of this article (as noticed by a referee), both conventions were used.

If we reduce everything to within-cycle differences first, then the random patient and cycle terms are eliminated, and only ${\mathit{\sf{\sigma}}}^{\sf{2}},{\mathit{\sf{\gamma}}}^{\sf{2}}$are relevant to calculating our estimates. We can have

2 $$\hat{\tau}=\frac{\sum_{i=1}^n{\sum}_{r=1}^{k_i}\frac{Y_{ir2}-{Y}_{ir1}}{Z_{ir2}-{Z}_{ir1}}}{\sum_{i=1}^n{k}_i}$$

as an estimate of $\mathit{\sf{T}}$. Since ${\mathit{\sf{Z}}}_{\mathit{\sf{ir}}\sf{2}}-{\mathit{\sf{Z}}}_{\mathit{\sf{ir}}\sf{1}}=\sf{1},-\sf{1}$ depending on whether A is given on the first occasion in a cycle or the second, this is simply the sum of all the within-cycle differences for treatment B minus treatment A divided by the total number of cycles. If we have the same number of cycles, *k*, per patient, this simplifies to

3 $$\hat{\tau}=\frac{\sum_{i=1}^n{\sum}_{r=1}^k\frac{Y_{ir2}-{Y}_{ir1}}{Z_{ir2}-{Z}_{ir1}}}{nk}.$$

What the appropriate variance of this estimator is depends on what we consider it is an estimate of, that is to say, what we consider *Τ* to be. For example, if we take it to be an estimate of the mean treatment effect for these patients, then this is fixed for the sample. We shall refer to this as the *local purpose*. We then have that the variance is

4 $$Var\left(\hat{\tau}\right)=\frac{2{\sigma}^2}{\sum_{i=1}^n{k}_i}.$$

Note, as discussed above, the appearance of the factor 2 because variances of within cycle differences have a contribution from each of two error terms.

In the balanced case where *k*_*i*_ = *k*, ∀ *i*, then we have

5 $$Var\left(\hat{\tau}\right)=\frac{2{\sigma}^2}{nk}.$$

On the other hand, if we take *Τ* to be the mean treatment effect in a population of patients from whom the patients studied may be taken to be a random sample, then we have

6 $$Var\left(\hat{\tau}\right)=\frac{\psi^2}{n}+\frac{2{\sigma}^2}{\sum_{i=1}^n{k}_i},$$

with, in the balanced case,

7 $$Var\left(\hat{\tau}\right)=\frac{\psi^2}{n}+\frac{2{\sigma}^2}{nk}.$$

We refer to this as the *global purpose*. Note that for the global purpose, (a) this estimator is only optimal in the unbalanced case or if *ψ*^2^ = 0, and (b) whether or not this is optimal, the variance for the global is only the same as for the local purpose if *ψ*^2^ = 0.

An alternative approach to estimation starts with the individual patient estimates,

8 $${\hat{\tau}}_i=\frac{\sum_{r=1}^{k_i}\frac{Y_{ir2}-{Y}_{ir1}}{Z_{ir2}-{Z}_{ir1}}}{k_i}.$$

For the global purpose, these have variances

9 $$Var\left({\hat{\tau}}_i\right)={\psi}^2+\frac{\sigma_d^2}{k_i},.$$

where ${\sigma}_d^2=2{\sigma}^2$, with the subscript *d* standing for difference.

These estimates may then be combined in a weighted sum to produce an estimate

10 $${\hat{T}}_{global}={\sum}_{i=1}^n{w}_i{\hat{\tau}}_i,$$

where

11 $${w}_i=\frac{\frac{1}{\psi^2+\frac{\sigma_d^2}{k_i}}}{\sum_{i=1}^n\left(\frac{1}{\psi^2+\frac{\sigma_d^2}{k_i}}\right)},$$

that is to say, with weights inversely proportional to the variance and summing to one. Note that (9), (10), and (11) define an estimate that has the same general form as a random effects meta-analysis estimator, the only practical difference being that *σ*^2^should be estimated globally, rather than individually patient by patient. The variance of (10) is given by

12 $$Var\left({\hat{T}}_{global}\right)=\frac{1}{\sum_{i=1}^n\frac{1}{Var\left({\hat{\tau}}_i\right)}}.$$

Note also that if *k*_*i*_ = *k*, ∀ *i*, *i* = 1⋯*n*, we have from (10) that ${\hat{T}}_{global}=\frac{\sum_{i=1}^n{\hat{\tau}}_i}{n}$ and from (12) that $Var\left({\hat{T}}_{global}\right)=\frac{Var\left(\hat{\tau}\right)}{n}$.

In the ‘Estimates of effects for individual patients’ section, the formula for shrunk estimates was given as

13 $$shrunk=w\times personal+\left(1-w\right) global.$$

If we assume that a suitably large number of patients have been studied, then the global estimate as a prediction for the long-term average may be assumed to have a variance of *ψ*^2^, whereas the local estimate for patient *i* may be assumed to have a variance of $\frac{2{\sigma}^2}{k_i}$. These two estimates should be weighed proportionately to the inverse of their variances, so we have

14 $$w=\frac{\psi^2}{\psi^2+\frac{2{\sigma}^2}{k_i}},1-w=\frac{\frac{2{\sigma}^2}{k_i}}{\psi^2+\frac{2{\sigma}^2}{k_i}}.$$

Since *w*is the weight for the personal element and *ψ*^2^ is the variation in the true treatment effect from patient to patient, we can see that, other things being equal, as this variation becomes more important, more weight is given to the global estimate. Similarly, since 1 − *w* is the weight for the global estimate, we can see that as the within patient variation *σ*^2^ gets larger, then more weight will be given to the global estimate, although this can be reduced by increasing the number of cycles *k*_*i*_ in which the patient is observed.

Finally, we have as a formula for the variance of the shrunk estimate,

15 $$Var(shrunk)=\frac{2{\psi}^2{\sigma}^2}{k_i{\psi}^2+2{\sigma}^2}.$$

Note that if we have no local information on patient *i* so that *k*_*i*_ = 0, we have that (15) is equal to *ψ*^2^, which, since we must rely on global information only, is to be expected. On the other hand, as *ψ*^2^ → ∞, we have that (15) $\to \frac{2{\sigma}^2}{k_i}$ which is the personal variance, which again is only to be expected, since the results from other patients contribute no information. In general, however, (15) is lower than either the global or the personal variance. Thus, an advantage of the shrunk estimate is the reduction in variance that it brings.

A further point to note is that the formula does not allow for uncertainty in the global estimate itself. The uncertainty in using the global estimate as a prediction of the effect for a given patient reflects the variation of the individual patient effects from the supposed true average global value. In practice, this global value itself is subject to uncertainty and, as a referee has pointed out, since the values from an individual also contribute to the global estimate, there will also be a small correlation between the two, which is also in practice ignored. In short, this approach works best when one has data from many patients. See also my paper on sample size determination [22] for further discussion.
